# Stereotactic body radiation therapy on abdominal-pelvic lymph node oligometastases: a systematic review on toxicity

**DOI:** 10.2340/1651-226X.2024.40681

**Published:** 2024-10-29

**Authors:** Lucy A. van Werkhoven, Eugenio Cammareri, Mischa S. Hoogeman, Remi A. Nout, Maaike T.W. Milder, Joost J.M.E Nuyttens

**Affiliations:** a Erasmus MC Cancer Institute, University Medical Center Rotterdam, Department of Radiotherapy, The Netherlands; b Department of Oncology and Hemato-Oncology, University of Milan, Milan, Italy

**Keywords:** SBRT, lymph node, oligometastases, toxicity, review

## Abstract

**Background and purpose:**

To review available data on toxicity during and/or after treatment of abdominal-pelvic lymph node oligometastases (A-P LN) with stereotactic body radiation therapy (SBRT) and to provide an overview of adverse events and its relation to dose or fractionation.

**Material and methods:**

For this systematic review, we searched MEDLINE, Embase, Web of Science Core Collection, and CINAH for studies published between the database inception and October 3rd, 2023. Inclusion criteria were (1) patients with 1–5 A-P LN oligometastases, (2) treatment with SBRT to a median prescribed dose of ≥55 Gy BED_10_, and (3) description of acute and/or late toxicity. There were no language or date restrictions.

**Results:**

A total of 35 studies, including 1,512 patients, were selected. Late grade 3 and 4 adverse events occurred in 0.6% and 0.1% of the patients treated for A-P LN oligometastases. All late adverse events grade ≥ 3 occurred after treatment of the tumor with a minimum BED_10_ of 72 Gy. Of the 11 patients with severe late toxicity, five patients were re-irradiated. Late grade 2 and 1 toxicity was reported in 3.4% and 8.3% of the patients. Acute toxicity grades 4, 3, 2, and 1 occurred in 0.1%, 0.2%, 4.4%, and 19.8% of the patients, respectively.

**Interpretation:**

SBRT for A-P LN oligometastases show low toxicity rates. Nearly 50% of late adverse events ≥ grade 3 were associated with re-irradiation.

## Introduction

Oligometastatic disease is an intermediate state of cancer spread between localized disease and widespread metastases, with no more than five metastases [1–4]. This intermediate state of cancer used to be treated with systemic treatments like chemotherapy. But, because of the limited amount of metastasis, metastatic-directed therapy (MDT) is increasingly used in the clinic [5]. MDTs, such as stereotactic body radiotherapy (SBRT), have the potential to achieve local control and offer improved disease free survival [6]. Multiple phase I/II studies have shown that a majority of treated metastasis (70% – 90% in many series) achieve local control [7]. Furthermore, the overall survival (OS) described after SBRT for oligometastatic disease was 92.3% and 79.2% at 1 and 2 years, and trials showed an improved 5-year OS for patients receiving SBRT and an improved progression-free survival in addition to the standard of care [8–10]. An additional benefit, beyond LC and long-term survival, is deferring the start of systemic treatment. Shahi et al. showed a 4-year chemotherapy-free survival of 69% following SBRT for abdominopelvic oligometastases [11]. By delaying systemic therapy, its side effects, with a possible negative impact on the quality of life, can be postponed as well [12].

Using SBRT, a high dose can be delivered to the tumor, while the dose to the organs at risk (OAR) can be minimized resulting in less normal tissue damage [13]. Especially in the abdominopelvic region, where oligometastases are frequently located in close proximity to radiosensitive organs, such as the bowel, this is of great importance. However, toxicity after SBRT remains a potential problem in this anatomical region with the additional challenge of highly mobile OAR.

For different anatomical regions, that is, adrenal, bone, liver, and lung, severe toxicity has been reported after SBRT. In the SABR-COMET trial, grade ≥ 2 toxicity was found in 29% of the patients, and three patients showed grade 5 toxicity (4.5%) [10]. These severe events occurred despite strict dose constraints and peer review of all radiation treatment plans, showing the importance of establishing dose-effect relationships. Multiple systematic reviews report on LC after SBRT of mixed lymph node sites. However, reports on toxicity after SBRT for abdominal-pelvic lymph node (A-P LN) oligometastases are scarce and fragmented, and to our knowledge, a systematic review on toxicity after SBRT on A-P LN has not been performed [14–16]. This review aims to evaluate toxicity during and/or after treatment of A-P LN oligometastases with SBRT and to provide an overview of reported adverse events.

## Methods

### Strategy

This review followed the Prisma guidelines (PRISMA Checklist in Supplementary Appendix 1) [17]. The search was performed on four databases (Medline, Embase, Web of Science, and CINAHL) until the 3rd of October 2023 (Supplementary Appendix 2). The search was arranged to identify publications reporting on toxicity in patients treated with SBRT on A-P LN. Therefore, we used the following search terms: SBRT, lymph nodes, and toxicity. The full line-by-line search for each database is available in the appendix (Supplementary Appendix 3). The search strategy employed no restrictions on language or date. The following inclusion criteria were used: (1) patients with 1–5 A-P LN oligometastases, (2) treated with SBRT with a median prescribed dose of ≥45 Gy equivalent dose in 2 Gy fractions α/β 10 (EQD2_10_) or ≥55 Gy biologically effective dose α/β 10 (BED_10_), and (3) acute and/or late toxicity for patients with A-P LN was described. Publications were excluded when (1) there were <10 patients with A-P LN oligometastases treated, and (2) the median follow-up was <6 months. Two researchers (LW, EC) independently screened titles and abstracts of every publication retrieved, and a third researcher (JN) was consulted in case no consensus was reached. Publications selected by both researchers were included in a full-text review.

### Data extraction and analysis

Data were extracted from the selected studies by two researchers, both residents in training at the radiation oncology, (LW, EC) independently; then the extracted data were compared, with any discrepancies being resolved through discussion. The following data were extracted from the publications: first author, year of publication, study design, number of patients, median follow-up, dosimetric details (total dose, number of fractions, and dose per fraction), dose constraints, number of patients with A-P LN oligometastases, number of A-P LN oligometastases treated, primary tumor, method for toxicity scoring (CTCAE or RTOG), number of patients with acute or late toxicity, the type of toxicity, and the use of concurrent chemotherapy. In case of a grade ≥ 3 toxicity, the following data were extracted: location of LN, volume of target LN, dose to OAR, time from treatment to severe event, and primary treatment with radiation. Not all publications reported all types and grades of toxicity. To avoid a bias, average incidence percentages were calculated by summing the total amount of patients with the reported type of toxicity divided by the total amount of patients reported in the correlating publications. In the publications that reported on multiple target sites, it is often unclear to which treated target site the toxicity can be attributed. This holds both for the incidence and the type of toxicity. For these cases, the incidence was not included in our quantitative assessment. In other publications, the authors did make a clear distinction in the incidence of toxicity per treated site but no distinction in the type of toxicities. In these cases, we have only included the incidence of toxicity and the type of adverse events if they could be clearly attributed to irradiation of the A-P region, for example, diarrhea. For toxicity, the CTCAE V3.0 and 4.0 were used most. The two versions had small differences: In case of asthenia as toxicity, this was classified as malaise or lethargy in CTCAE V3.0 and as fatigue in V4.0. Enterocolitis in V3.0 corresponded with enteritis in V4.0. Urinary tract pain from V3.0 corresponded with pain in V4.0. Some toxicity grades differed between the two CTCAE versions; liver enzyme cut-off value differed; dyspnea grades 3 and 4 were not entirely consistent with each other; incontinence grades 3 and 4 differed partly; and ileus grade 1 did not exist in V3.0. Furthermore, acute toxicities were defined as adverse events occurring within 3 months after SBRT, and late toxicities were those occurring after 3 months.

### Quality assessment

For quality assessment of etiology and harm-finding studies, the Newcastle-Ottowa scale (NOS) for cohort studies, recommended by The Cochrane Methods Prognosis group, was used [18]. The NOS was developed to assess the quality of nonrandomized studies. We used a modified 6-point NOS since only one study with two cohorts was included in this review, no points were accredited for the comparability of cohorts. A score of 5–6 points was rated as good, 3–4 points as intermediate, and 2 or less as poor quality. Studies were scored by two researchers (LW, EC) independently, then the scores were compared, with any discrepancies being resolved through discussion.

### Statistics

Statistical analyses were performed using Microsoft Excel 2016. Toxicity was assessed on a per-study basis and subsequently aggregated. The aggregated data were evaluated according to the total amount of patients in all included studies. An interquartile range (IQR) was calculated using IBM SPSS statistics (Version 28.0.1.0 (142))

## Results

### Eligible studies and quality assessment

**Figure 1:**
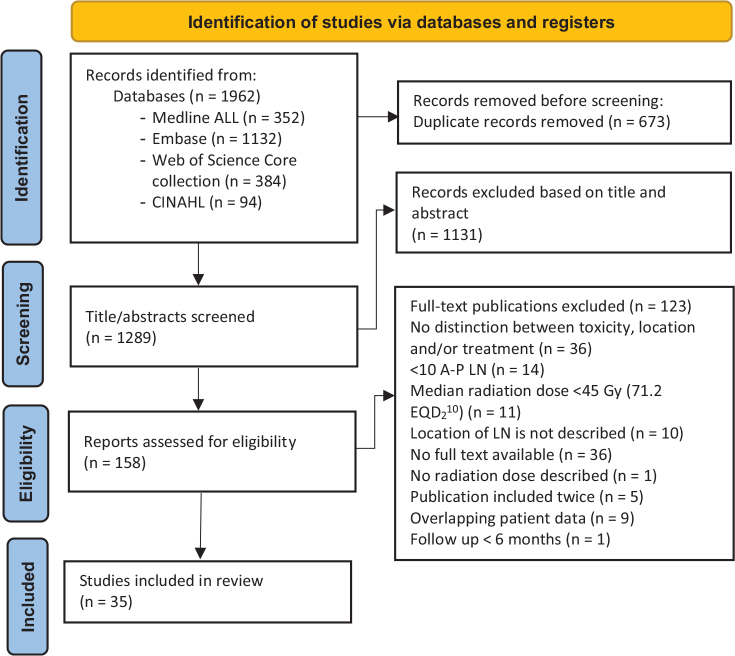
PRISMA flowchart [17]. Abbreviations: LN: lymph node; A-P LN: abdominal-pelvic lymph nodes.

The database search resulted in 1,962 publications. After the removal of 673 duplicates, 1,289 publications remained and were screened on title and abstract. Based on the title and abstract 1,131 studies were excluded, and 158 reports remained for full-text review, resulting in 35 studies that were included in this systematic review. Reasons of exclusion are listed in Figure 1. Of the 35 studies, 26 were retrospective studies, seven were prospective studies, and two combined retrospective and prospective studies. The median number of included patients per publication was 37 (range 11–101). The median follow-up was 21 months (range 10–42). Among the reviewed studies, 17 studies exclusively addressed patients with A-P LN, while 18 studies included other lymph node locations or different metastases. Primary tumors were prostate cancer in 16 reports, various tumors in 14, gastrointestinal (GI) tumors in 3, and hepatocellular carcinoma and cervix carcinoma were the primary tumor in one report, respectively. The median physical prescribed dose was available in only 20 studies and ranged from 24 to 48.5 Gy. The median number of fractions was described in 16 studies and ranged from 1 to 12 fractions. The median dose per fraction ranged from 4 to 24 and was reported in 13 publications. The CTCAE V4 toxicity classification was used in 23 studies. Concurrent treatment with chemotherapy during SBRT was reported in three studies and occurred 19.2%, 6.1%, and 3% of the patients [23, 26, 31]. A cutoff between acute and late toxicities of 3 months after start of radiotherapy was used in 12 studies. Cozzi et al. defined toxicity as late toxicity when reported ≥6 months after start of radiotherapy while Park et al. used a 6-week cutoff [25, 43]. Park et al. described the time interval between radiotherapy and the occurrence of late grade ≥ 3 for all patients; if this time interval was < 3 months, the toxicity was classified as acute within our review. 21 studies did not provide a definition for acute and/or late toxicity. An overview of the study characteristics can be found in Table 1. Table 2 shows the score per item of the quality assessment. The median score was 3 (Table 1). The different toxicity grades were not reported in all publications, for example, grade 3 and 4 acute toxicities were reported in 33 out of 35 publications, while acute grade 1 was reported in only 16 out of 35 publications (Table 3).

**Table 1:** Characteristics of included studies.

Author	Study design	N	N with A-P LN	Primary tumor	Median FU (months)	Median total dose (range)	Median number of fractions (range)	Median d/f (range)	Toxicity classification	QA score
Alsuhaibani, 2019 [19]	Retrospective	21	11	GI	17	n.a. (30–60)	n.a. (3–5)	n.a. (n.a.)	CTCAE V4.0	3
Barney, 2012 [20]	Retrospective	47	13	Various	12	45 (20–60)	5 (1–5)	10 (n.a.)	CTCAE V3.0	4
Bignardi, 2011 [21]	Retrospective	19	19	Various	12	45 (36–45)	6 (6)	n.a. (6–7.5)	CTCAE V3.0	3
Bouman, 2017 [22]	Retrospective	43	34	Prostate	31	n.a. (30–35)	n.a. (3–5)	n.a. (7–10)	n.a.	3
Burkon, 2020 [12]	Retrospective	90	57	Various	35	n.a. (27–45)	n.a. (3–8)	n.a. (5–15)	CTCAE (version n.a.)	3
Caivano, 2023 [23]	Retrospective	174	82	Various	NA	36 (14–76)	n.a. (1–8)	n.a. (4–23)	CTCAE V4.4	3
Corvò, 2013 [24]	Retrospective	36	36	Various	28	35 (12–50)	5 (2–10)	n.a. (4–9)	CTCAE V4.0	3
Cozzi, 2022 [25]	Retrospective	74	74	Prostate	31	40 (33–40)	5 (3–5)	8 (8–11)	CTCAE V4.0	3
Cuccia, 2023 [26]	Retrospective	69	66	Prostate	16	35 (30–40)	5 (3–6)	n.a. (n.a.)	CTCAE V4.0	3
Detti, 2015 [27]	Retrospective	30	30	Prostate	12	n.a. (24–36)	n.a. (1–5)	n.a. (6–24)	CTCAE V4.0	3
Franzese, 2017 [28]	Retrospective	26	26	Prostate	29	40 (25–45)	6 (4–6)	n.a. (n.a.)	CTCAE V4.0	3
Franzese, 2017 [29]	Retrospective	35	35	CRC	15	n.a. (30–45)	n.a. (6–13)	n.a. (3–7.5)	CTCAE V3.0	3
Franzese, 2016 [30]	Retrospective	71	71	Various	18	45 (45)	6 (6)	7.5 (7.5)	CTCAE V4.0	3
Franzese, 2020 [31]	Prospective	52	52	Various	24	48 (48)	4 (4)	12 (12)	CTCAE V4.0 & RTOG/EORTC	5
Gawish, 2023 [32]	Retrospective	17	17*	Prostate	16.6	48 (30–60)	12 (5–20)	4 (3–8)	n.a.	3
Ingrosso, 2017 [33]	Retrospective	40	39	Prostate	24	n.a. (12–50)	n.a. (1–5)	n.a. (5–12)	RTOG/EORTC criteria	4
Kang, 2010 [34]	Retrospective	59	30	CRC	32	42 (35–51)	3 (3)	n.a. (12–17)	CTCAE V2.0	4
Kneebone, 2018 [35]	Prospective	57	39	Prostate	16	n.a. (30–50)	n.a. (1–5)	10 (10)	CTCAE V4.0	5
Kutuk, 2022 [36]	Retrospective	96	52	Various	10	48.5 (30–60)	5 (3–15)	n.a. (n.a.)	CTCAE V4.0	2
Lepinoy, 2019 [37]	Retrospective	62	35	Prostate	42	36 (30–66)	n.a. (n.a.)	7.5 (2–15)	CTCAE V4.0	4
Loi, 2018 [38]	Retrospective	23	23	Prostate	22	24 (24)	1 (1)	24 (24)	CTCAE V4.0	3
Loi, 2018 [39]	Retrospective	91	89	Various	23	n.a. (40–48)	n.a. (5–6)	n.a. (7–9)	CTCAE V4.0	3
Matoba, 2020 [40]	Retrospective	15	15	HCC	18	n.a. (45–49.5)	n.a. (6–9)	n.a. (5.5–7.5)	CTCAE V4.0	4
Nicosia, 2022 [41]	Prospective	63	63	Prostate	17	35 (14–40)	n.a. (n.a.)	n.a. (5–21)	CTCAE V5.0	6
Ost, 2016 [42]	Retrospective	72	72	Prostate	36	n.a. (24–50)	n.a. (3–10)	n.a. (5–10)	CTCAE V4.0	3
Park, 2015 [43]	Retrospective	85	83	Cervix	20	39 (27–51)	n.a. (3–10)	13 (n.a.)	CTCAE V4.0	4
Pasqualetti, 2016 [44]	Prospective	29	17	Prostate	12	n.a. (24–27)	n.a. (1–3)	n.a. (9–24)	CTCAE V4.0	5
Pezzulla, 2021 [45]	Prospective	38	38	Prostate	27	n.a. (20–50)	n.a. (1–5)	n.a. (9–24)	CTCAE V4.0	6
Regnery, 2022 [46]	Prospective	26	26	Various	10	n.a. (25–40)	n.a. (3–7)	n.a. (5–9)	CTCAE V5.0	5
Shahi, 2020 [11]	Retrospective	51	48	Various	22	35 (25–40)	5 (5)	7 (5–8)	CTCAE V4.0	4
Siva, 2018 [47]	Prospective	33	13	Prostate	NA	20 (20)	1 (1)	20 (20)	CTCAE V4.0	6
Wang, 2016 [48]	Retrospective	22	22	Various	33	39 (21–51)	5 (3–8)	8 (5–13)	CTCAE V4.0	4
Werensteijn, 2021 [49]	Prospective/retrospective	90	90	Prostate	21	n.a. (30–35)	n.a. (3–6)	n.a. (6–10)	RTOG/EORTC criteria	5
Yang, 2022 [50]	Prospective/retrospective	101	101	Various	11	40 (25–50)	5 (n.a.)	8 (5–10)	CTCAE V5.0	5
Yeung, 2017 [51]	Retrospective	18	11	Various	34	n.a. (30–60)	n.a. (4–8)	n.a. (5–8)	CTCAE V4.0	3
**Median:**		**47**	**37**		**21**					3

Abbreviations: n.a.: not available; *N*: number of patients; A-P LN: abdominal pelvic lymph nodes; FU: follow up; d/f: dose per fraction; QA: quality assessment, CRC : colorectal cancer, HCC: hepatocellular carcinoma.

* 17 patients were treated for a total of 28 lesions (21 LN and 8 bone metastasis). Unclear if all patients had at least one LN.

**Table 2:** Quality assessment of all included studies (*n* = 35) using Newcastle-Ottowa Quality Assessment Scale (NOS).

	*N*	%
Selection		
Representativeness of the exposed cohort	35	100%
Selection of the nonexposed cohort*	n.a.	n.a.
Ascertainment of exposure	35	100%
Demonstration that outcome of interest was not present at the start of the study	9	26%
Comparability of cohorts*		
Study controls for most important factor	n.a.	n.a.
Study controls for any factor	n.a.	n.a.
Outcome		
Assessment of outcome	16	46%
Follow up long enough (minimum 1 year)	30	86%
Adequacy of follow up (≥90% of all patients	8	23%
Scores		
6 points	3	9%
5 points	6	17%
4 points	8	23%
3 points	17	49%
2 points	1	3%

Abbreviations: n.a.: not applicable; *N*: the number of studies that fulfill this criterion.

For each item of the NOS, one point could be obtained.

* Since there was only one study with two cohorts, no points were accredited for the comparability of cohorts.

**Table 3:** Overview of toxicities per study.

Study	N pts A-P LN	Acute toxicity in grade:				Late toxicity in grade:			
		1	2	3	4	1	2	3	4
Alsuhaibani (2019) [19]	11	n.a.	2	0	0	n.a.	0	0	0
Barney (2012) [20]	13	n.a.	n.a.	0	0	n.a.	n.a.	0	0
Bignardi (2011) [21]	19	4	0	0	0	1	0	1	0
Bouman (2017) [22]	34	1	2	0	0	0	0	0	0
Burkon (2020) [12]	57	n.a.	n.a.	0	0	0	0	0	0
Caivano, (2023) [23]	82	n.a.	n.a.	0	0	0	0	0	0
Corvò (2013) [24]	36	23	0	0	0	0	0	0	0
Cozzi (2022) [25]	74	0	0	0	0	0	0	0	0
Cuccia (2023) [26]	66	n.a.	n.a.	0	0	n.a.	n.a.	0	0
Detti (2015) [27]	30	0	1	0	0	1	0	0	0
Franzese (2017) [29]	35	2	3	0	0	n.a.	n.a.	n.a.	n.a.
Franzese (2017) [28]	26	5	0	0	0	0	0	0	0
Franzese (2016) [30]	71	10	2	0	0	0	0	0	0
Franzese (2020) [31]	52	4	0	0	0	0	0	0	0
Gawish (2023) [32]	17	n.a.	n.a.	0	0	n.a.	n.a.	0	0
Ingrosso (2017) [33]	39	n.a.	1	0	0	n.a.	0	1	0
Kang (2010) [34]	30	n.a.	n.a.	0	2	n.a.	n.a.	n.a.	n.a.
Kneebone (2018) [35]	39	n.a.	n.a.	0	0	n.a.	n.a.	0	0
Kutuk (2022) [36]	52	n.a.	n.a.	0	0	n.a.	n.a.	0	0
Lepinoy (2019) [37]	35	0	2	1	0	2	12	2	0
Loi (2018) [38]	23	2	0	0	0	0	0	0	0
Loi (2018) [39]	89	26	13	0	0	5	5	0	0
Matoba (2020) [40]	15	8	1	0	0	0	0	0	0
Nicosia (2022) [41]	63	0	0	0	0	0	0	0	0
Ost (2016) [42]	72	n.a.	n.a.	n.a.	n.a.	12	3	0	0
Park (2015) [43]	83	n.a.	n.a.	1	0	0	9	2	2
Pasqualetti (2016) [44]	17	n.a.	0	0	0	n.a.	0	0	0
Pezzulla (2021) [45]	38	n.a.	n.a.	0	0	n.a.	n.a.	0	0
Regnery (2022) [46]	26	9	2	0	0	n.a.	n.a.	n.a.	n.a.
Shahi (2018) [11]	48	n.a.	n.a.	1	0	1	0	0	0
Siva (2018) [47]	13	n.a.	n.a.	0	0	n.a.	n.a.	0	0
Wang (2016) [48]	22	n.a.	n.a.	0	0	0	0	0	0
Werensteijn (2021) [49]	90	48	3	0	0	18	8	0	0
Yang (2022) [50]	101	n.a.	n.a.	n.a.	n.a.	53	4	3	0
Yeung (2017) [51]	11	n.a.	3	0	0	n.a.	0	0	0
Total toxicity		**142**	**35**	**3**	**2**	**93**	**41**	**9**	**2**
Reported in studies (*n*)*		16	20	33	33	21	25	32	32
Total patients in studies		718	796	1356	1356	1122	1200	1438	1438
% patients with toxicity		**19.8**	**4.4**	**0.2**	**0.1**	**8.3**	**3.4**	**0.6**	**0.1**

Abbreviations: N pts A-P LN: number of patients treated on abdominal-pelvic oligometastases; n.a.: not available.

* Number of studies reporting the incidence of this specific type and grade of toxicity.

### Late toxicities

A late grade ≥ 3 toxicity was found in 11 (0.8%) patients and was reported in 32 of the 35 studies. Late grade 3 toxicity was found in 0.63% and grade 4 toxicities in 0.14% of the patients, respectively. This percentage ranged from 0% to 5.7% among the different studies with an interquartile range (IQR) of 0%-0%. Five of the 11 patients were treated with re-irradiation for an infield recurrence. The status of combination therapy for these individual cases was unknown. Table 3 shows the number of adverse events per study. Gastrointestinal (GI) grade ≥ 3 adverse events occurred in 7 patients, and genitourinary (GU) grade ≥ 3 adverse events were diagnosed in 4 patients. The median physical dose to the tumor in the patients who had late grade ≥ 3 GI and GU toxicity was BED_10_ 79.2 Gy (range 72–86.5 Gy) and 75.6 (range 72–79.2 Gy), respectively. This toxicity occurred after a median time of 5.7 months (range 3.4–11.8 months) and 18.4 months (range 5.7–20.7), respectively. Details of the adverse events can be found in Table 4. No grade 5 toxicity was reported in patient cohorts included in this review.

**Table 4:** Late grade ≥ 3 toxicity.

Case	Study	Grade	Type toxicity	Time (months)	BED_10_ (Gy)”	Total Dose (Gy)”	d/f (Gy)	*N* fractions	Previous radiation (total dose)	D_max_ OAR
	**Gastro-intestinal toxicity**									
1	Park. 2015 [43]	4	Recto-vaginal fistula	5.7	86.5	38	12.7	3	Yes (50.4 Gy)	n.a.
2	Park, 2015 [43]	4	Recto-vaginal fistula	6.3	79.2	36	12	3	Yes (50.4 Gy)	n.a.
3	Park, 2015 [43]	3	Ileus	3.7	79.2	36	12	3	Yes (n.a.*)	n.a.
4	Yang, 2022 [50]	3	Colonic hemorrhage	3.4	72	40	8	5	No	36 Gy
5	Ingrosso, 2017 [33]	3	Small bowel obstruction	11.8	n.a.	n.a.	n.a.	n.a.	n.a.	n.a.‡
6	Bignardi, 2011 [21]	3	Obstruction	n.a.	81.1	45	7.7	6	No	n.a.
7	Lepinoy, 2019 [37]	3	Gastrointestinal event	n.a.	n.a.	39*	n.a.	n.a.	n.a.	n.a.
			**Median**	**5.7**	**79.2**					
	**Genitourinary toxicity**									
8	Park, 2015 [43]	3	Urethral stricture	20.7	79.2	36	12	3	Yes (50.4)	n.a.
9	Yang, 2022 [50]	3	Ureteral stenosis	10.1	72	40	8	5	Yes (60 Gy)	50.6 Gy
10	Yang, 2022 [50]	3	Ureteral stenosis	18.4	72	40	8	5	No	44.8 Gy
11	Lepinoy, 2019 [37]	3	Genitourinary event	n.a.	n.a.	n.a.†	n.a.	n.a.	n.a.	n.a.
			**Median**	**18.4**	**75.6**					

Abbreviations: n.a.: not available; N: Number; d/f: dose per fraction; Time: time from treatment to adverse event.

‡ 13 cc of the bowel received a dose of 6 Gy per fraction (total 24 Gy, BED_3_ 72 Gy);

* Previously treated with radical concurrent chemoradiation therapy and intracavity radiotherapy;

† median radiation dose in the study was 39 Gy; “Prescribed dose to target.

Late grade 2 toxicity was reported in 25 publications for 41 (3.4%) patients. The number of patients who reported a late grade 2 or higher toxicity was 4.3%. Late grade 1 toxicity was found in 93 patients (8.9%) and was described in 21 publications. In 23 publications, the late grade ≤ 2 toxicity was classified. GU and GI toxicities were reported in 26 and 21 patients, respectively. Details of grade 1 and 2 late toxicities are shown in Table 5.

**Table 5:** Type of acute and late grade ≤2 toxicity.

Acute grade 1 and 2 toxicity	*N*	Late grade 1 and 2 toxicity	*N*
General	79	General	12
Fatigue	53	Pain	7
Pain	12	Fatigue	5
Asthenia	10	Gastrointestinal	21
Anorexia	3	Diarrhea	3
Skin reaction	1	Rectal hemorrhage	1
Gastrointestinal	89	Proctitis	1
Nausea/vomiting	36	Not specified	17
Diarrhea	15	Genitourinary	26
Constipation	3	Dysuria	1
Dysphagia	5	Not specified	25
Acute enteritis	4	Other	4
Liver enzyme elevation	3	Sciatic nerve pain	1
Gastritis	2	Vertebral fracture	1
Duodenal ulcer	1	Not specified	2
Rectal Hemorrhage	1		
Not specified	19		
Genitourinary	14		
Dysuria	4		
Cystitis	1		
Urinary incontinence	1		
Erectile dysfunction	1		
Not specified	7		
Other	9		
Acute hematologic toxicity	3		
Dyspnea	1		
Not specified	5		

### Acute toxicities

Acute grade 4 toxicity was reported in 33 out of 35 publications and found in 2 (0.1%) patients. Acute grade 3 toxicity occurred in 3 (0.2%) patients and was reported in 33 publications. Within these 5 acute grades ≥ 3 toxicities, there were 4 GI events and 1 GU event. One of these five patients was treated with 51 Gy in 3 fractions (BED_10_ 138 Gy) on a pelvic lymph node and developed a rectal perforation requiring a rectal anastomosis [34]. The second patient was treated with 48 Gy in 3 fractions (BED_10_ 125 Gy) to a para-aortic lymph node, experienced an intestinal obstruction that required surgery [34]. The third patient required hospitalization due to SBRT-related nausea and dehydration after receiving 30 Gy in 5 fractions (BED_10_ 48 Gy) to a solitary peri-portal lymph node; this patient had multiple comorbidities [11]. The fourth patient had a grade 3 enterocolitis 2.7 months after treatment of a left common iliac lymph node with 32 Gy in 5 fractions (BED_10_ 53 Gy) [43]. Finally, Lepinoy et al. reported an acute grade 3 genitourinary event, though specific details were not provided [37]. So, the events occurred in patients who were treated with a high median BED_10_ of 89 Gy (range 48–138 Gy) [11, 34, 43] were re-irradiated [43] or had comorbidity [11].

Acute grade 2 toxicity was reported in 20 out of 32 studies and occurred in 35 (4.4%) patients. Acute grade 1 toxicity was reported in 16 studies and was found in 142 (19.8%) patients. The toxicity was classified in 19 publications. GI toxicity was reported in 89 patients, general toxicity like fatigue, pain, etc. was reported for 79 patients, and 14 patients developed GU grade ≤2 acute events. Details on grade ≤2 are described in Table 5.

### Constraints for organs at risk

The applied constraints for organs at risk were described in 21 out of the 35 studies and varied widely. In six studies, dose constraints to OAR were based on 2010 recommendations of the American Association of Physicists in Medicine [52]. Using a fractionation schedule with 5 fractions, they advise a D_max_ point dose of 38 Gy for the colon, rectum, and bladder, 35 Gy for the small bowel, and 32 Gy for the duodenum and stomach. Details on constraints used in the publications can be be found in Table 6. To enable comparison, constraints in 5 fractions are reported here, as they were most common in the selected publication.

**Table 6:** Summary of constraints used in the publications. Only the constraints in 5 fractions are reported here.

OAR	American Association of Physicists in medicine Task group [52]* used by [11, 22, 25, 38, 39, 44]	Chang et al. [53] and Hoyer et al. [54] used by [20]	AAPM Task group [52], S.S. Lo [55] and de Pooter et al. [56] used by [12]	Used by [35]	Used by [36]	Used by [37]	UK consensus by Hanna et al. [57] used by [46]	Used by [50]	Timmermans et al. [58] used by [19]
Duodenum	D_max_ 32 Gy	D_max_ 42	D_max_ 35 Gy		D_0.03cc_ ≤ 40 Gy	D_max_ 36 Gy	D_0.5cc_ < 35 Gy	D_0.03cc_ ≤ 35 Gy	D_max_ 32 Gy
	(BED_3_ 100)	(BED_3_ 160)	(BED_3_ 117)		(BED_3_ 147)	(BED_3_ 122)	(BED_3_ 117)	(BED_3_ 117)	(BED_3_ 100)
	D_5cc_ < 18 Gy	D_5cc_ ≤ 37 Gy	D_5cc_ < 18 Gy		D_0.5cc_ ≤ 36 Gy	D_0.5cc_ ≤ 30 Gy		D_0.5cc_ ≤ 33 Gy	
	D_10cc_ <12.5 Gy	D_15cc_ ≤ 32.5 Gy	D_10cc_ < 12.5 Gy		D_1cc_ ≤ 33 Gy				
		D_30cc_ ≤ 20 Gy			D_5cc_ ≤ 30 Gy				
Small bowel	D_max_ 35 Gy	D_max_ 42 Gy	D_max_ 35 Gy	D_2cc_ <25Gy	D_0.03cc_ ≤ 40 Gy	D_max_ 36 Gy	D_0.5cc_ < 35 Gy	D_0.03cc_ ≤ 35 Gy	D_max_ 35 Gy
	(BED_3_ 117)	(BED_3_ 160)	(BED_3_ 117)		(BED_3_ 147)	(BED_3_ 122)	(BED_3_ 117)	(BED_3_ 117)	(BED_3_ 117)
	D_5cc_ <19.5 Gy	D_5cc_ ≤ 37 Gy	D_5cc_ < 19.5 Gy		D_0.5cc_ ≤ 36 Gy	D_0.5cc_ ≤ 30 Gy		D_0.5cc_ ≤ 33 Gy	
		D_15cc_ ≤ 32.5 Gy			D_1cc_ ≤ 33 Gy				
		D_30cc_ ≤ 20 Gy			D_5cc_ ≤ 30 Gy				
Colon	D_max_ 38 Gy	D_max_ 42 Gy	D_max_ 38 Gy		D_0.03cc_ ≤ 40 Gy	D_max_ 36 Gy	D_0.5cc_ < 35 Gy	D_0.03cc_ ≤ 35 Gy	D_max_ 38 Gy
	(BED_3_ 134)	(BED_3_ 160)	(BED_3_ 134)		(BED_3_ 147)	(BED_3_ 122)	(BED_3_ 117)	(BED_3_ 117)	(BED_3_ 134)
	D_20cc_ < 25 Gy	D_5cc_ ≤ 37 Gy	D_20cc_ < 25 Gy		D_0.5cc_ ≤ 33 Gy	D_0.5cc_ ≤ 30 Gy		D_0.5cc_ ≤ 33 Gy	
		D_15cc_ ≤ 32.5 Gy							
		D_30cc_ ≤ 20 Gy							
Rectum	D_max_ 38 Gy	D_max_ 42 Gy	D_max_ 38 Gy		D_0.1cc_ ≤ 38 Gy	D_max_ 36 Gy	D_0.5cc_ < 34 Gy	D_0.1cc_ ≤ 36.25 Gy	D_max_ 38 Gy
	(BED_3_ 134)	(BED_3_ 160)	(BED_3_ 134)		(BED_3_ 134)	(BED_3_ 122)	(BED_3_ 111)	(BED_3_ 124)	(BED_3_ 134)
	D_20cc_ < 25 Gy	D_5cc_ ≤ 37 Gy	D_20cc_ < 25 Gy		D_1cc_ ≤ 36 Gy	D_0.5cc_ ≤ 30 Gy		D_1cc_ ≤ 38.06 Gy	
		D_15cc_ ≤ 32.5 Gy			D_5cc_ ≤ 34 Gy				
		D_30cc_ ≤ 20 Gy			D_10cc_ ≤ 33 Gy				
					D_20cc_ ≤ 25 Gy				
Bladder	D_max_ 38 Gy	D_max_ 42 Gy	D_max_ 38 Gy		D_0.1cc_ ≤ 38 Gy	D_max_ 36 Gy	D_0.5cc_ < 38 Gy	D_0.1cc_ ≤ 36.25 Gy	D_max_ 38 Gy
	(BED_3_ 134)	(BED_3_ 160)	(BED_3_ 134)		(BED_3_ 134)	(BED_3_ 122)	(BED_3_ 134)	(BED_3_ 124)	(BED_3_ 134)
	D_15cc_ < 18.3 Gy	D_5cc_ ≤ 37 Gy	D_15cc_ < 18.3 Gy		D_1cc_ ≤ 36 Gy	D_0.5cc_ ≤ 30 Gy		D_1cc_ ≤ 38.06 Gy	
		D_15cc_ ≤ 32.5 Gy			D_15cc_ ≤ 33 Gy				
		D_30cc_ ≤ 20 Gy							
Stomach	D_max_ 32 Gy	D_max_ 42 Gy	D_max_ 32 Gy		D_0.03cc_ ≤ 40 Gy	D_max_ 36 Gy		D_0.03cc_ ≤ 35 Gy	D_max_ 32 Gy
	(BED_3_ 100)	(BED_3_ 160)	(BED_3_ 100)		(BED_3_ 147)	(BED_3_ 122)		(BED_3_ 117)	(BED_3_ 100)
	D_10cc_ < 18 Gy	D_5cc_ ≤ 37 Gy	D_10cc_ < 18 Gy		D_0.5cc_ ≤ 33 Gy	D_0.5cc_ ≤ 30 Gy		D_0.5cc_ ≤ 33 Gy	
		D_15cc_ ≤ 32.5 Gy							
		D_30cc_ ≤ 20 Gy							

Abbreviations: OAR: organ at risk. The BED_3_ was calculated for the D_max,_ and if not available for the near D_max_ (D_≤1cc_).

* max point dose.

## Discussion

To our knowledge, this is the first systematic review providing an overview of toxicity specifically related to the treatment of A-P LN oligometastases. The main finding of our review, of 35 studies, is that toxicity rates after treatment of A-P LN oligometastases are relatively low, for example, late grade ≥ 3 severe events were seen in less than 1% of the patients. Reviews on toxicity after SBRT of mixed lymph node sites show that less than 5% of the patients experienced late grade ≥ 2 toxicity, and incidence rates of grade ≥ 3 and grade 5 toxicities were 2.0% and 0.2%, respectively [15, 59]. We found comparable results with 4.3% late grade ≥ 2 toxicity. When evaluating the advantages of MDT with SBRT, the improvement in overall survival and disease-free survival seems proportional with the low incidence of side effects. For example, Deodato et al. conducted a review on patients treated with SBRT on LN oligometastases with mixed lesion sites and reported a median OS of 29 months (range 18–43 months) [15]. More evidence of the benefits of SBRT is shown by Philips et al., as they reported a significant improvement of the median progression-free survival (not reached vs. 5.8 months) in patients treated with SBRT for oligometastatic prostate cancer [9]. When focusing on grade ≥ 3 toxicity only, our review showed an average incidence close to 1%, which corresponds to values previously reported in the literature. However, the 5 publications included in this review that actually report late grade ≥ 3 toxicity, show an incidence ranging from 2.5% to 5.3%. All patients in these 5 publications with a late severe adverse event were treated with a minimum of BED_10_ of 72 Gy to the tumor. The D_max_ to the OAR was only described in 3 of these cases: the D_max_ for the bowel was BED_3_ 122.4 Gy and for the ureter BED_3_ 178.6 Gy and 221.3 Gy [50]. Our analysis of acute toxicity showed grade ≥ 2 toxicity in 4.5% of the patients. A review from Ponti et al. showed acute and/or late grade ≥ 2 toxicity in 5.6% of patients with oligo-recurrent prostate cancer limited to lymph nodes at pelvic and extra-pelvic sites [16].

We observed, in our review, that five of the 11 severe adverse events occurred in patients who underwent re-irradiation [43, 50]. Of these five patients, four were reported by Park et al., who included 89 LN oligometastases [43]. In this study, re-irradiation took place in 15.4% of the para-aortic LNs and 64.5% of the pelvic LN, and this resulted in four late adverse events of grade 3 or higher. Details on the previous radiation dose were described for 3 of the cases and resulted in a summed BED_10_ of 139–146 Gy. The occurrence of severe toxicity upon re-irradiation is not unexpected, given the high cumulative treatment dose. This corresponds to findings in the review of Jereczek-Fossa et al., 2015, the researchers stated that high toxicity after SBRT in LN oligo metastatic patients was observed, particularly in re-irradiation cases [60].

The performed quality appraisal of literature included in this systematic review showed a median score of 3 points, which can be interpreted as intermediate quality. This can be explained by the inclusion of mainly retrospective studies, which are generally of lower quality compared to prospective studies. Only one publication scored 2 points and was assigned as poor quality.

One of the reasons to treat people with MDT is to postpone systemic therapy and thereby postponing systemic side effects that may negatively impact quality of life (QoL). Remarkably, in the reviewed publications, only two articles reported QoL [47, 49], while toxicity was mostly reported by the radiation oncologists, using CTCAE. Although our search terms did not include QoL, we did expect to find more articles that describe QoL in addition to toxicity. As QoL after SBRT of A-P LN is an important outcome for this group of patients, we recommend to include QoL as an endpoint in future studies.

Our review has several limitations. The number of subjects with A-P LN metastasis in the publications included in our review is limited (median 37, range 11–101). In addition, most of the publications included report on retrospective studies (74%). It is known that reporting toxicity retrospectively can lead to information bias and an underestimation of the toxicity, especially for grade 1–2 toxicity [61]. Furthermore, none of the 35 publications reported all incidence of grade 1 to 4 acute and late toxicities; however, we attempted to avoid bias due to the calculation of the incidence by summing the total amount of patients with the reported type of toxicity divided by the total amount of patients reported in the corresponding publications. Moreover, there are more factors besides treatment dose that may cause an increased risk for toxicity, for example, concurrent chemotherapy, comorbidities, and in field re-irradiation. In the majority of the studies, these data were not available but the three studies that included patients who were treated with concurrent chemotherapy did report 0% grade ≥ 3 acute and late toxicities.

An additional limitation in our study is the large heterogeneity in the way the publications report their study parameters and findings. Dosimetric data were limited or missing in some publications. Furthermore, the available dosimetric data showed that studies are heterogeneous in terms of the median total dose (20–48 Gy), the median number of fractions (1–12), and the median dose per fraction (4–24 Gy), resulting in a wide range of BED_10_. There is still an ongoing debate regarding the exact definitions of SBRT. In this study, we included patients who, according to the authors, were treated with SBRT. Additionally, we applied a criterion requiring a minimum median prescribed dose of 45 Gy EQD2 in an attempt to filter out non-SBRT results in mixed studies. In one study, the reported toxicity was derived from stereotactic treatments and nonstereotactic treatments [37].

Due to the heterogeneity of several aspects in the publications, it is hard to compare different publications. One of these aspects is the differences in the applied dose constraints. Given that dose constraints for a 5-fraction radiation schedule were most documented, we only reported on 5-fraction constraints to provide a good comparison. Constraints for the colon and small bowel ranged from a (near) D_max_ of 117–160 BED_3_ for 5 fractions. This range was even larger for the duodenum (100–160 BED_3_) and the rectum (111–160 BED_3_). Barney et al. used a D_max_ of 42 Gy(BED_3_ 160) for all viscous hollow organs at risk, which is the highest D_max_ used compared to other included publications [20]. However, they did not report any acute or late grade ≥ 3 adverse events while Lepinoy et al. and Shahi et al. did report severe adverse events using lower D_max_ constraints ranging from BED_3_ 117-124 Gy for various OAR [11, 37]. In our review, we found a total of 4 severe late adverse events involving the GU tract [37, 43, 50]. None of the 3 studies that reported these adverse events reported about constraints for the ureters. However, Yang et al. reported that, after their study, a dose volume constraint to the ureter was introduced (V45 < 0.03 cm^3,^ BED_3_ 128) [50]. As the incidence of severe toxicity was low, we consider the constraints as given in Table 6 adequate. The constraints of the AAPM task group were most frequently used although some researchers, for example Barney et al., used higher constraints [20, 52]. It would be insightful if these higher constraints would be validated in larger prospective studies.

Another aspect that adds to the heterogeneity and uncertainty in our data is the nature of A-P LN; they are often surrounded by highly radiosensitive and mobile OAR. This causes multiple challenges. The delivered dose can deviate from the intended, protocolized dose for both target and OAR. First, in some cases, the dose to the target has to be compromised to obey the OAR constraints due to the close proximity of the OAR to the target. These deviations are usually not recorded separately in publications. Second, due to the motion of the OAR, the initial planned doses to the OAR may not correspond with the delivered dose. Both may lead to a deviation of the protocolized dose constraints. Online verification, or even adaptation, could be used to reduce the dose to the OAR and/or increase the dose to the target and bridge the gap between intended and delivered dose [50]. In addition, online adaptive treatments can give a more accurate estimate of the delivered dose [21, 43].

## Conclusion

SBRT for A-P LN oligometastases comes with a low incidence of toxicity. The occurrence of severe adverse events is rare and often associated with re-irradiation. Despite the variation in the published dose constraints across different studies, they appear to be practical in clinical use to minimize toxicity, but further refinement is warranted.

## Supplementary Material

Stereotactic body radiation therapy on abdominal-pelvic lymph node oligometastases: a systematic review on toxicity

## Data Availability

All data generated and analyzed during this study are included in this publication (and its Supplementary Material).

## Credit author statement

Lucy van Werkhoven: Methodology, formal analysis, investigation, writing – original draft.

Joost Nuyttens: conceptualization, formal analysis, writing – review and editing, supervision.

Maaike Milder: conceptualization, writing – review and editing, supervision.

Eugenio Cammareri: Investigation, writing – review and editing.

Remi Nout: Conceptualization, writing – review and editing, supervision.

Mischa Hoogeman: conceptualization, writing – review and editing, supervision.
